# Next-generation sequencing: a follow-up of 36,913 singleton pregnancies with noninvasive prenatal testing in central China

**DOI:** 10.1007/s10815-020-01977-2

**Published:** 2020-10-23

**Authors:** Wan Lu, Ting Huang, Xin-Rong Wang, Ji-Hui Zhou, Hui-Zhen Yuan, Yan Yang, Ting-Ting Huang, Dan-Ping Liu, Yan-Qiu Liu

**Affiliations:** Prenatal Diagnosis Center, Jiangxi Maternal and Child Health Hospital, Nanchang, 330006 Jiangxi China

**Keywords:** Noninvasive prenatal testing (NIPT), Chromosomal abnormalities, Prenatal diagnosis, Performance

## Abstract

**Purpose:**

To evaluate the noninvasive prenatal testing (NIPT) results of 36,913 cases in Jiangxi province of central China and explore its application value in prenatal screening and diagnosis.

**Methods:**

This retrospective analysis included 36,913 singleton pregnant women who underwent NIPT because of moderate-/high-risk pregnancy or voluntary requirements between January 2017 and December 2019 in our hospital. Chromosomal abnormalities such as trisomies 21, 18, and 13 (T21, T18, T13) and sex chromosome aneuploidies (SCAs) were judged by standard Z-score analysis. Positive NIPT results were confirmed by amniocentesis and karyotyping. Pregnancy outcomes were followed up via telephone interview.

**Results:**

A total of 1.01% (371/36,913) positive cases were detected by NIPT, comprising 137, 46, 31, and 157 cases of T21, T18, T13, and SCAs, respectively. A total of 116 of T21, 27 of T18, 13 of T13, and 51 of SCAs were confirmed to be true positive; all normal cases that had been followed up were verified to be true negative. The NIPT sensitivity in T21, T18, T13, and SCAs was 100.00% individually, whereas the specificity was 99.94% (36,488/36,509), 99.95% (36,579/36,598), 99.95% (36,594/36,612), and 99.72% (36,472/36,574), respectively. Furthermore, the negative predictive values of T21, T18, T13, and SCAs were all 100%, while the positive predictive values were 84.67%, 58.70%, 41.94%, and 33.33%, respectively.

**Conclusion:**

NIPT is highly sensitive and has a low false positive rate in testing clinically significant fetal aneuploidies of general reproductive women. However, this technique cannot substitute for amniocentesis and karyotyping, and detailed genetic counseling is also essential for the high-risk group of NIPT.

## Introduction

Chromosomal abnormalities, which include trisomy 21 (T21), trisomy 18 (T18), trisomy 13 (T13), and sex chromosome aneuploidies (SCAs), are the main causes of birth defects, especially in pregnancies of numerous older women in China, considering their “two-child policy” [1]. These chromosomal diseases often result in mental retardation and growth or developmental delay, accompanied by severe deformity of facial features, limbs, or other aspects. Unfortunately, no curative treatment is currently available for such birth defects. Therefore, an accurate and effective method that detects fetuses with high chromosomal aneuploidy risk is necessary to reduce birth defects. Serological screening is the traditional method of prenatal screening, but it has a low detection rate, high false positive rate (FPR), and low positive predictive value (PPV) [2]. Other prenatal diagnosis methods, such as amniocentesis, chorionic villus sampling, and umbilical cord blood sampling, are generally accurate, but the high risk of procedure-related miscarriage may limit their clinical application [3, 4].

Since cell-free DNA (cfDNA) was detected in the peripheral blood of pregnant women by Lo YM in 1997 [5], and with the rapid development of high-throughput sequencing technology in recent years, noninvasive prenatal testing (NIPT) has been gradually developed to analyze cfDNA and deduce the presence of fetal chromosomal abnormalities through amplification, high-throughput sequencing technology, and bioinformatics processing [6]. Compared with serological screening, sonographic screening, and other traditional interventional prenatal diagnosis methods, NIPT is noninvasive, has high sensitivity, and can avoid the risk of abortion, infection, or injury caused by invasive interventional operations [7]. Therefore, NIPT is rapidly being employed to detect the fetal chromosome aneuploidies of T21, T18, T13, and SCAs around the world. The American College of Medical Genetics and Genomics (ACMG), along with several other professional associations, has issued statements and guidelines suggesting that NIPT is a screening test to identify pregnancies at risk for common autosomal aneuploidies [8–11]. In addition, a continuously updated meta-analysis of Gil MM showed that screening by analysis of cfDNA in maternal blood (including NIPT) in singleton pregnancies could detect > 99% of fetuses with T21, 98% of fetuses with T18, and 99% of fetuses with T13 at a combined FPR of 0.13%, and the performance of cfDNA testing for T21 in twin pregnancy is similar to that reported in singleton pregnancy [12, 13]. Since NIPT was proposed as a screening test in China in 2011, several studies from multiple centers have reported that it can offer a perfect performance for detecting chromosomal abnormalities of fetus [14–16]. A recent study of 31,515 singleton pregnancies in southeastern China showed a high sensitivity and specificity for T21, T18, T13, and SCAs, and the positive predictive values were high for T21 and T18 and moderate for T13 and SCAs [16]. Therefore, NIPT has been implemented into public healthcare systems as either a first-line test or a supplement to the existing prenatal screening; more and more pregnant women are willing to choose NIPT [17–19].

However, large-scale NIPT performance in the general population of central China remains uninvestigated. In this study, we aimed to retrospectively analyze and follow up 36,913 pregnant women who received NIPT to assess the accuracy and feasibility of NIPT in prenatal testing and find the possible reasons for false-positive and false-negative results.

## Materials and methods

### Study subjects

Singleton pregnant women who came to Prenatal Diagnosis Center of Jiangxi Maternal and Child Health Hospital between January 2017 and December 2019 were recruited for this study. All the participants had undergone pretest counseling and provided an informed written consent before blood sample collection. Based on maternal age and other risk factors, we categorized subjects into four groups as follows: advanced maternal age (≥ 35 years) with high risk, advanced maternal age with low risk, normal maternal age (< 35 years) with high risk, and normal maternal age with low risk. In this study, “high risk” was defined as a high/moderate risk of Down syndrome, the presence of ultrasound soft markers, or a history of previous Down syndrome pregnancy. In contrast, the absence of any of these risk factors defined “low risk”.

### Sample collection and preparation

Approximately 5 mL of peripheral blood was collected from the pregnant women in tubes primed with ethylenediaminetetraacetic acid. Within 96 h after blood collection, plasma was isolated from the blood samples in accordance with the two-step centrifugation protocol [20]. The samples were first centrifuged at 1600*g* under 4 °C for 10 min. Then, the supernatant was collected and re-centrifuged at 16,000*g* for 10 min to remove the remaining white blood cells or cell debris. Thereafter, plasma samples were frozen at – 80 °C.

### cfDNA extraction and DNA sequencing

**Fig. 1:**
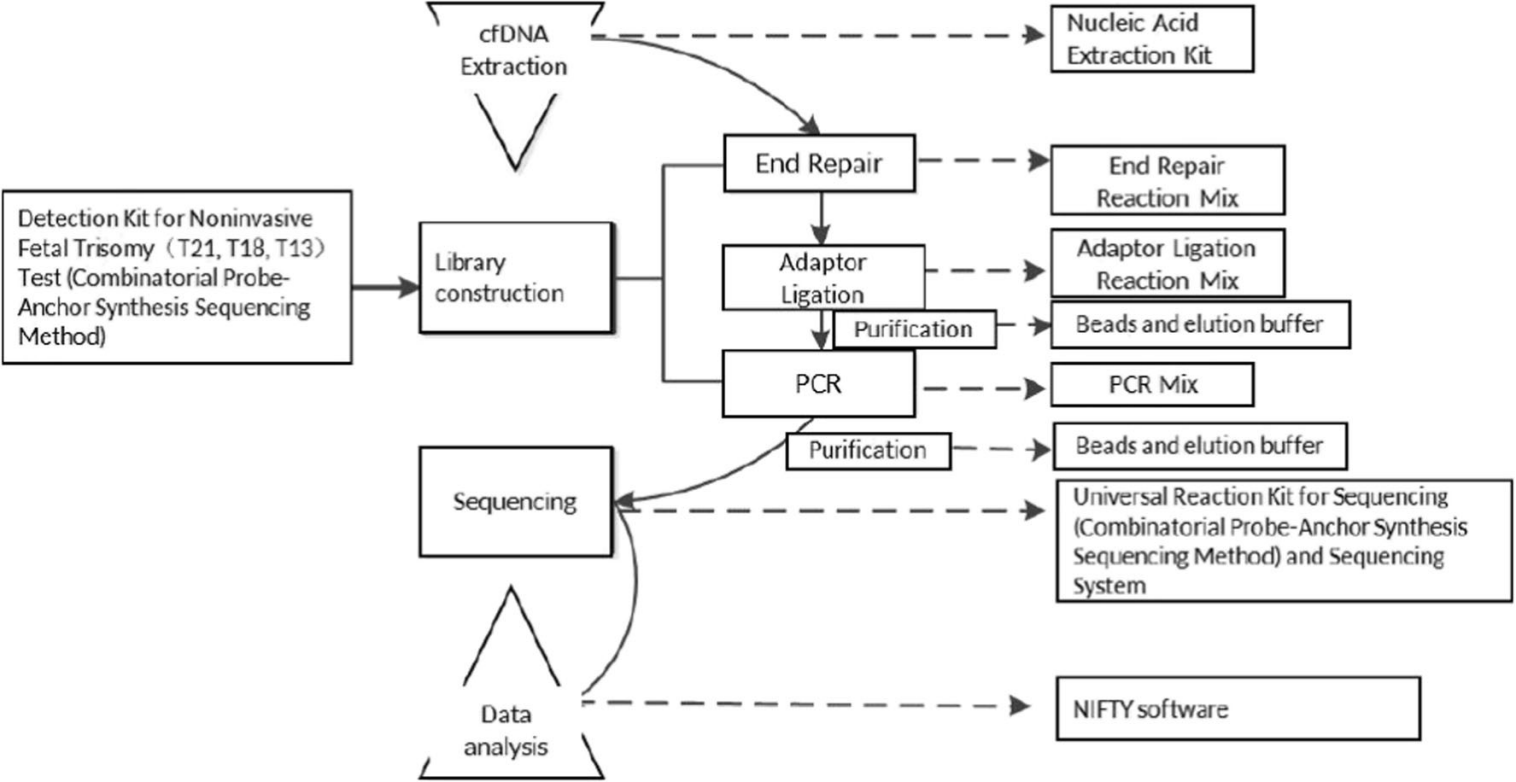
The workflow of detection process detection kit for noninvasive fetal trisomy (T21, T18, T13) test (combinatorial probe-anchor synthesis sequencing method) utilizes combinatorial probe-anchor synthesis sequencing method to detect cell-free fetal DNA in maternal plasma samples. After the specific adaptor ligation, amplification, purification, and sequencing of cell-free DNA, the effective reads of each chromosome are obtained through sequencing alignment. Through comparison with cut-off values, fetal chromosomal aneuploidy can be detected.

Plasma cfDNA was extracted from the isolated plasma samples according to the manufacturer’s instructions of Nucleic Acid Extraction Kit (Beijing Genomics Institution, BGI, China), and the concentration of cfDNA was measured with a Qubit™ Fluorometer (Thermo Fisher Scientific, USA). Combinatorial probe-anchor synthesis sequencing method was used for DNA sequencing; 96 libraries were sequenced with 45-cycle single-end sequencing on BGISEQ-500 platforms. After the specific adaptor ligation, amplification, and sequencing of cfDNA, we obtained the effective reads of each chromosome by sequencing alignment. Through comparison with cut-off values, fetal chromosomal aneuploidies (T21, T18, and T13) could be identified [21]. Figure 1 illustrates the detection process based on the BGI user manual.

### Prenatal diagnosis and pregnancy follow-up

When the Z-score of chromosomes 21, 18, or 13 of the sample to be detected is ≥ 3, it is considered positive. Every participant received genetic counseling after NIPT screening. Subjects with positive NIPT results were suggested to be verified with invasive testing for prenatal diagnosis. To obtain information about neonatal outcome and newborn growth, we followed up all participants via telephone interviews 1 month after the expected date of delivery.

### Data and statistical analysis

To characterize NIPT performance, we calculated the sensitivity, specificity, FPR/false negative rate (FNR), and predictive values through the following formulas. Sensitivity = TP/(TP + FN), specificity = TN/(FP + TN), FNR = FN/all cases, FPR = FP/all cases, PPV = TP/(TP + FP), and negative predictive value (NPV) = TN/(FN + TN). TP, FN, TN, and FP stand for true positive, false negative, true negative, and false positive, respectively. All statistical data were analyzed using the statistical software SPSS version 19.0, difference of positive rate between advanced maternal age and normal age was tested for statistical significance using Chi-square test, and *P* < 0.05 was considered statistically significant.

## Results

### Patient characteristics and clinical indication

Between January 2017 and December 2019, 37,006 singleton pregnant women were recruited to the study. The median maternal age was 29 years (range 18 to 54 years), and the median gestational age was 17 + 4 weeks (range 12 to 32 weeks). However, 306 (0.83%) cases required repeat blood sampling because of borderline Z-score or low fetal fraction, and 213 of them obtained effective NIPT results. Ultimately, 36,913 samples that obtained informative results were included in this study while 93 (0.25%) were excluded.

In total, we identified 371 positive NIPT results from the 36,913 pregnancies, yielding an overall abnormal rate of 1.01%. The advanced maternal age (≥ 35 years) with high-risk group had the highest rate (1.88%) of chromosomal abnormalities, whereas the normal age (< 35 years) with low-risk group had the lowest (0.69%). The positive rate was significantly higher in advanced maternal age than in normal age (1.30% vs. 0.90%, *P* < 0.05) (Table 1).

**Table 1:** Detection of fetal aneuploidies in different indications

Indications	Detected number	Positive number	Positive rate (%)
Advanced maternal age	9516	124	1.30
With high risk	1118	21	1.88
With low risk	8398	103	1.23
Normal maternal age	27397	247	0.90
With high risk	12575	145	1.15
With low risk	14822	102	0.69
Total	36913	371	1.01

### Chromosomal abnormality and prenatal diagnosis

**Fig. 2:**
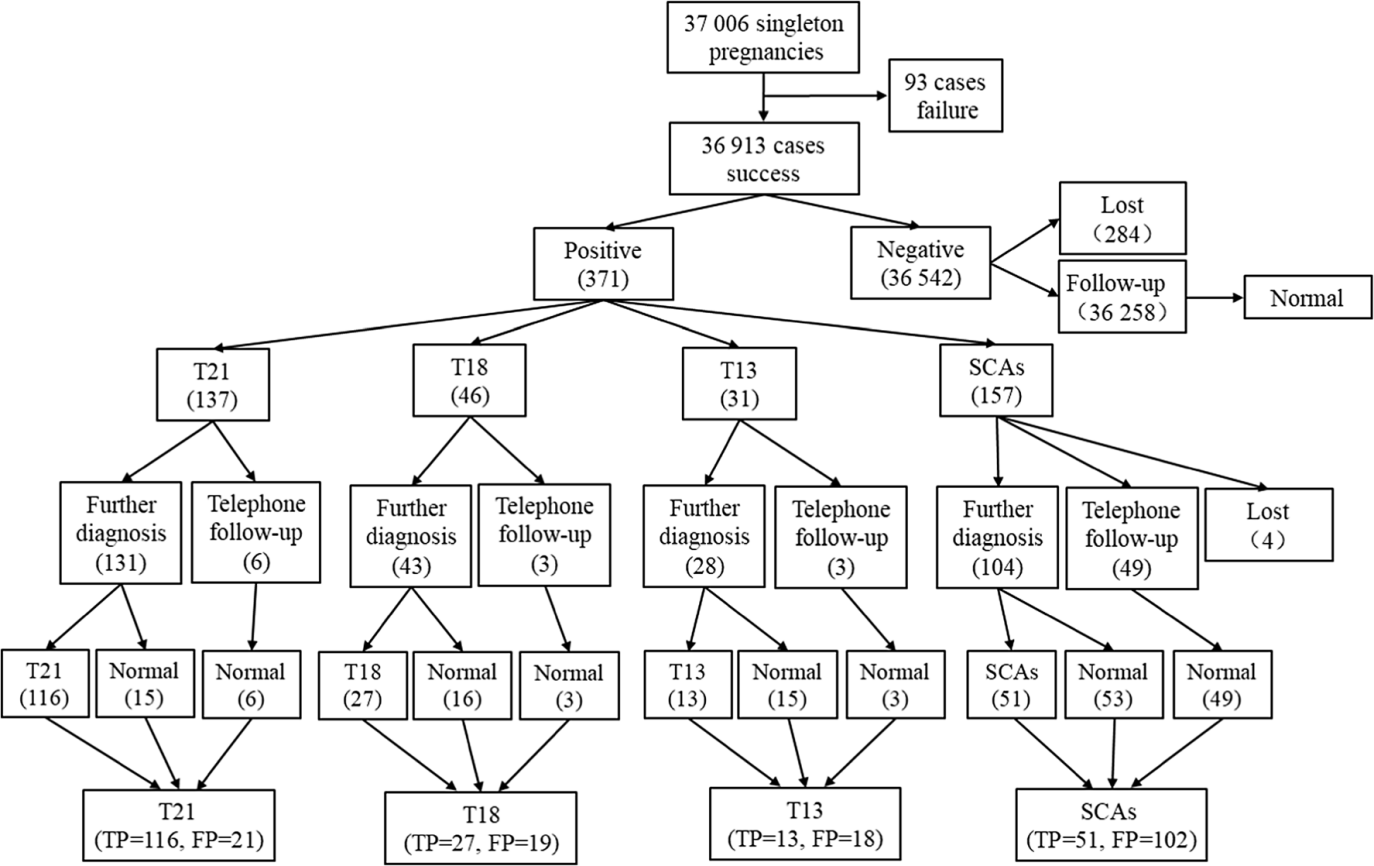
Flowchart of NIPT results and follow-up

Among the 371 positive cases identified by NIPT, the most common chromosomal abnormality was SCAs (157 cases, 42.32%), followed by T21 (137 cases, 36.93%), T18 (46 cases, 12.40%), and T13 (31 cases, 8.35%). Positive NIPT results were verified by amniocentesis and karyotyping. After genetic counseling, 277 pregnancies underwent prenatal diagnostic testing, and 190 cases were confirmed to be true positive (105, 25, 12, and 48 cases of T21, T18, T13, and SCAs, respectively) (Table 2). Of the 94 pregnancies who refused to take the prenatal diagnostic testing, four cases were lost to follow-up, 29 cases had received karyotyping of their newborns, and 17 cases had chromosome abnormalities (11, 2, 1, and 3 cases of T21, T18, T13, and SCAs, respectively) (Table 3). Figure 2 presents a complete flowchart of NIPT results and follow-up.

**Table 2:** Fetal karyotypes of NIPT positives

Prenatal diagnosis (number)	Fetal karyotypes	Number
T21 (117)	47,XN,+21	96
	47,XN,+21/46,XN	6
	46,XN,rob(13;21),+21	2
	46,XN,rob(14;21),+21	1
	46,XN	12
T18 (40)	47,XN,+18	25
	46,XN	15
T13 (26)	47,XN,+13	12
	46,XN	14
SCAs (94)	45,X	5
	47,XXX	10
	47,XXY	17
	47,XYY	3
	45,X/46,XX	2
	47,XXX/46,XX	4
	46,X,del(X)	2
	45,X/46,X,i(X)(q10)	1
	46,X,add(X)	3
	46,X,psu dic(X)(p11.2)	1
	46,XN	46

**Table 3:** Follow-up of all NIPT positives

	T21	T18	T13	SCAs
Amniocentesis karyotypes	117	40	26	94
Abnormal	105	25	12	48
Normal	12	15	14	46
Newborn karyotypes	14	3	2	10
Abnormal	11	2	1	3
Normal	3	1	1	7
No karyotyping	6	3	3	49
Total	137	46	31	153^a^

^a^Four cases were lost to follow-up

### Performance of NIPT for detecting fetal chromosomal aneuploidies

Based on the results of amniocyte karyotyping and newborn follow-up, 116 out of 137 (84.67%) cases for T21, 27 out of 46 (58.70%) cases for T18, 13 out of 31 (41.94%) cases for T13, and 51 out of 153 (33.33%) cases for SCAs were confirmed to be true positive; all normal cases of NIPT were verified to be true negative, except 284 cases, which had been lost to follow-up. To evaluate the performance of NIPT, we calculated key parameters such as sensitivity, specificity, FPR, FNR, PPV, and NPV. The sensitivity and NPV for T21, T18, T13, and SCAs were all 100%, whereas the FN and FNR were 0. The overall specificity for detecting these four chromosomal anomalies ranged from 99.72 to 99.95%, and the PPV for T21, T18, T13, and SCAs was 84.67%, 58.70%, 41.94%, and 33.33%, respectively (Table 4).

**Table 4:** Performance of NIPT for detecting fetal chromosomal aneuploidies

Chromosome abnormality	TP	FP	TN	FN	Sensitivity (%)	Specificity (%)	FPR (%)	FNR (%)	PPV (%)	NPV (%)
T21	116	21	36,488	0	100	99.94	0.06	0	84.67	100
T18	27	19	36,579	0	100	99.95	0.05	0	58.70	100
T13	13	18	36,594	0	100	99.95	0.05	0	41.94	100
SCAs	51	102	36,472	0	100	99.72	0.28	0	33.33	100

## Discussion and conclusion

Chromosomal abnormalities, with an increasing incidence of approximately 1.5 in 100 births in China, constitute one of the major causes of birth defects [22, 23]. A valid and accurate prenatal screening or diagnostic method is necessary for the prenatal diagnosis, in order to reduce the incidence of birth defects and improve the quality of the population. In 2016, the American Society of Medical Genetics suggested that NIPT could be used as a first-line screening method for T21, T18, and T13 in all pregnant women [8]. Since then, NIPT has been widely used for the prenatal screening of T21, T18, T13, and SCAs, and an increasing number of studies have successfully evaluated the performance of NIPT around the world [8–10, 14–16]. However, a large-scale clinical study on the efficacy of NIPT in Jiangxi province of central China is still unavailable. Therefore, we hope that the present study, which includes 36,913 cases, can provide a satisfactory performance report to settle this issue.

This study identified 371 high-risk patients by NIPT, among which 306 received further diagnosis and 207 were confirmed, and 21, 19, 18, and 102 cases of T21, T18, T13, and SCAs respectively, were confirmed to be false positive. Meanwhile, except for 284 cases which were lost to follow-up, no false-negative result was found. Overall, the sensitivity and specificity of NIPT for detecting chromosomal abnormalities were high, reaching 100% and 99.94% for T21, 100% and 99.95% for T18 and T13, and 100% and 99.72% for SCAs, consistent with those reported in previous studies in China [18, 24]. Furthermore, the PPVs of T21, T18, T13, and SCAs were 84.67%, 58.70%, 41.94%, and 33.33%, respectively, indicating a high PPV for T21, a moderate PPV for T18, and a low PPV for T13 and SCAs. Hence, some of the positive cases turned out to be false positive. Although our PPV results are consistent with previous reports worldwide [25–27], several reasons contribute to having false-positive results, including maternal chromosomal abnormality [28, 29], confined placental mosaicism [30, 31], vanishing twin [32], maternal copy number variations [33], fetal fraction [34], and so on. In addition, a short-term telephone follow-up to ascertain whether the newborns are normal or not is not entirely reliable and can also contribute to false-positive results. In our study, 17.5% (65 cases of 371) of pregnant women with positive NIPT results refused to undergo chromosomal diagnosis, excluding 4 cases lost to follow-up, 61 cases were identified to have normal newborns merely by telephone follow-up in 3 months after delivery, which were too early to judge the chromosomal abnormalities, especially for SCAs. Moreover, the lower guanine/cytosine deoxyribonucleotide content of chromosome 13 can cause the sequencing bias and may relate to the lower PPV of T13 [35].

Serological screening for trisomies based on maternal serum-free β-human chorionic gonadotrophin (β-HCG), pregnancy-associated plasma protein-A (PAPP-A), and α-fetoprotein (AFP) levels is still widely used in China. These biochemical markers, combined with maternal age, are usually used for screening for Down syndrome and can achieve a detection rate of 70–80% but a PPV of almost 10% [19]. Compared with NIPT for detection of T21 in our study, the sensitivity and specificity of serological screening seemed unsatisfactory. If all of the samples in our study had been detected by serological screening, more T21 fetuses would have been missed, and more false-positive cases would have been detected, resulting in additional amniocentesis; thus, more pregnancies would likely have been lost after invasive operation. Therefore, NIPT has shown more accuracy than serological screening, and ACMG have proposed NIPT to be offered for pregnant women of different ages.

With the full liberalization of the two-child policy and the popularization of late marriage and late childbirth, the proportion of older pregnant women (≥ 35 years old) in China is increasing. Older pregnant women are vulnerable to the influence of internal and external environment, ovarian function degeneration, and egg aging. Consequently, they are at an increased risk of chromosomal variation in the embryonic stage [36, 37]. Thus, pregnancy at an advanced age has become an independent risk factor for chromosomal aneuploidy formation [38]. According to the latest guideline about the management of prenatal diagnosis technology in China, primiparous women beyond 35 years old should undergo an invasive diagnostic procedure, and NIPT should be used modestly. In our study, 9516 pregnant women with an advanced age (≥ 35 years) still selected NIPT as the prenatal screening method, and 124 of them obtained positive NIPT results (1.30%), which were significantly higher than those with a normal maternal age (0.90%), suggesting a statistically significant difference (*P* < 0.05). Pregnant women with an advanced age can also acquire a satisfactory NIPT performance, indicating that NIPT can be offered as a prenatal screening method for any maternal age, along with the stress brought on by the two-child policy to the prenatal diagnosis system in China. Through NIPT application, almost 72% of older pregnant women were prevented from undergoing invasive tests, thereby leading to a 91% reduction in the number of procedure-related pregnancy loss [39]. Thus, adequate genetic counseling is necessary for the screening of older pregnant women, and NIPT can be used as the main screening method to detect and diagnose fetal chromosomal abnormalities early and effectively reduce the birth of children with defects.

However, this study has several limitations that should be clarified. Although no false-negative result was found in our study, 284 negative cases of NIPT were lost to follow-up. Additionally, observing false-negative results merely by the appearance of newborns is difficult. Although the International Society for Prenatal Diagnosis recommends NIPT as the first-line prenatal screening test for all pregnant women, most of our subjects still selected NIPT as the second-line screening test, main reason may be because of its high cost. Nevertheless, the market price of NIPT has already reduced to nearly 1800RMB (US$266) in our city; it is still higher than serological screening (approximately $31), but clearly less expensive than amniocentesis karyotype (approximately $960). Thus, more and more parents will choose NIPT as their first-line prenatal screening test in the future. In addition, when the content of cfDNA is below 4.0%, NIPT may fail or result in false-positive and false-negative results [40]. Our study initially examined 37,006 single fetal pregnancies, and 93 of them failed to obtain effective results because the fetal concentration was below 4.0%, with a failure rate of 0.25%, which is lower than that reported in previous studies [41–43]. Therefore, combining invasive prenatal diagnosis is necessary to avoid false positives. Overall, with the increase in the quantity of test specimens and the information analysis method of optimizing of large-scale genome sequencing technology applied in fetal chromosomal aneuploidy, NIPT will have a wide prospect of clinical application.

In summary, NIPT has great advantages in accuracy, specificity, and acceptance of pregnant women, especially in T21 screening; thus, it can effectively avoid birth defect occurrence and improve the quality of the birth population. Notably, NIPT is a prenatal screening method but not a substitute for prenatal diagnosis. Establishing a perfect genetic counseling system is crucial, and clinicians should correctly understand the advantages and limitations of NIPT technology in testing before and after detailed genetic counseling for pregnant women. In clinical application, this study, which focuses on the individual situation of pregnant women, fully informs the detection range and limitations of NIPT in pregnant women according to the need of clinical application. However, further research is required to determine how to integrate the data of NIPT prenatal screening in various regions into the national gene bank to improve the accuracy of NIPT prenatal screening and reduce the testing cost.

## Data Availability

Completely transparent.
